# The Best Bread

**Published:** 1891-11

**Authors:** 


### THE BEST BREAD.

Dr. B. F. Richardson, F. R. S., in his *Guild of Life*, says: Many people think that because it is fashionable to eat the whitest bread, therefore the whitest bread is the best for food. There cannot be a greater delusion. The coarser or brown bread—the bread that is made from what is known as whole meal—is the proper bread. I see, day by day, many evils which arise from the custom of having for food the white bread. The mothers are so thin that they look like starved mothers; and they are so, while the babes are absolutely wretched and starved, because the mothers are. I try, under these circumstances, to make the mothers understand that this white bread is the worst food they can take, and, in the end, the dearest; and if I can get them to believe this, and to change the food for something cheaper and better, it is astonishing how much healthier both the mother and the infant soon become.

The grain of wheat has a complicated and beautiful structure. If we look at the white inner part, which composes the geater portion of the grain, under the microscope, we observe that it is composed of a vast number of cells, each crowded with round masses called starch granules. The starch granules of wheat vary very much in size. Of the smallest there would be required 10,000, and of the largest, 900, placed end to end, to extend one inch in length. On the outer part of the wheat grain, but underlying the husk, is a layer of large cells, of a slightly yellow color; these are called the gluten cells. A little gluten and other albuminous matter are found in the starch cells, but it is in the outer layer that this matter exists in the greatest abundance. Gluten, albume n, and similar bodies are classified by the chemist as proteids. They are popularly called flesh formers, because they serve to build up the muscles and tissues of the body. These bodies all contain nitrogen as an essential constituent. At one end of the grain is the germ, wherein lies that most mysterious thing we call the life. Covering and protecting the parts we have briefly described is the testa, or branny covering. The outer part is silicious and indigestible, but the inner part is rich in serviceable mineral matters and flesh formers.

We will consider the disadvantages of using white bread. First, from 100 pounds of good white wheat we obtain only 73 pounds of fine flour; thus there is a great waste of human food, and the cost of the flour is increased. If the "tailings" are redressed and the "middlings" reground by the miller, thus producing a less white flout, the quantity will be raised to 80 pounds, but still there is a loss of 20 pounds.

Fine flour is poorer in albuminoids, or flesh formers, in fat and in mineral matters, than seconds flour.

The bran is very rich in mineral matters and bone formers, consequently only brown bread should be given to children, as they require phosphate of lime for the building up of their rapidly-growing bones.

The grains—of which wheat is the most valuable—stand above all other foods in their richness in compounds of phosphoric acid and in general high nutritive value, and they are the most satisfying and sustaining. It is, therefore, important that this, our most trusted food, should be of the best quality and not the white, excessively dressed and overfermented product supplied by most bakers.

